# Success in Surgery; a Collection of Clinical Facts

**Published:** 1843-07

**Authors:** 


ART. II.—*Du Bonheur en Chirurgie, Recueil de Faits Cliniques.* Par J. MOULINIÉ, Ex-Chirurgien en chef de l'Hôpital de Bourdeaux, Prof. de Clinique Chirurgicale, &c.—*Paris,* 1842. 8vo, pp. 224.

*Success in Surgery; a Collection of Clinical Facts.* By J. MOULINIÉ, late Principal Surgeon to the Hospital of Bourdeaux, Professor of Clinical Surgery, &c.—*Paris,* 1842.

THE conceit evinced in the title of this book is enhanced by a presumptuous and vain-glorious preface. "Demand of Fame," says M. Moulinié, "speak to my pupils, question my nurses, converse with men in practice who have attended my clinique, all will declare that my hospital practice has been distinguished by a rare degree of success. If such has not been the case with others, doubtless it is because others committed mistakes,"—which, of course, the all-fortunate M. Moulinié could not do. Still the book itself is not so bad as these self-glorifications would lead us to expect. The writer certainly does not appear to have duly estimated the serious nature of the subject, which he has chosen. The tone of the book is frivolous and jocose; the quantity of new matter small, and the proofs of the principles desired to be established so deficient, as to be quite insufficient to convince any disbeliever. The author, in fact, has done a great act of injustice to himself; for sufficient hints occur here and there in the book to show that he has removed numerous abuses which existed on his introduction to the hospital, has been most diligent in the discharge of his duties, and in his own words has considered "humanity as the first duty of the surgeon."

To the English reader there is little new or of sufficient interest to attract attention, the best part being that which is devoted to the account of the author's success in operations on varicose veins. The varicose vein to be operated on having been chosen, the following proceeding is recommended from the writer's own experience:

"A fold of skin parallel to the vein, and at some distance above the varicose part, having been selected, an incision is made across this fold, down to the cellular tissue round the vein, disturbing the part by dissection as little as possible. An eye-probe armed with silk or a fine thread is then passed under the vein, by which the vein is tied. An incision is often made across the vein a little above the ligature in order to prevent any inflammation of the vein spreading by continuity along it. The skin is then brought together by strapping, in order to protect the vein from the contact of air, as much as possible." (p. 112.)

This plan appears from the accounts given by the pupils who have observed the practice of M. Moulinié, to have been unattended with bad results, and to have been very successful.

An interesting case of atrophy of one leg, existing from birth, is related (p. 81,) which was amputated at the request of the patient below the knee. The parts were all wasted, fatty and thin. The arteries could not be found, a little blood only flowing from the situation of the posterior tibial artery, when a ligature was placed; but even then it was doubtful whether it included this vessel. No secondary bleeding occurred, and the case did well.

